# Enhancing Facial Esthetics in a Complete Denture Patient Having Sunken Cheeks With a Hollow Fixed Cheek Plumper: A Case Report

**DOI:** 10.7759/cureus.27539

**Published:** 2022-07-31

**Authors:** Shreya Colvenkar, Suman Pathipaka, Devarakonda Siva Santosh Babu, Kasukurthi Vijay Kumar, Rathod Prakash

**Affiliations:** 1 Prosthodontics, MNR Dental College and Hospital, Sangareddy, IND; 2 Oral and Maxillofacial Surgery, MNR Dental College and Hospital, Sangareddy, IND

**Keywords:** facial aesthetics, edentulous, fixed, esthetics, cheek plumper, denture

## Abstract

In today’s world, facial esthetics play a vital role in social and professional life. Prosthodontic rehabilitation deals not only with the replacement of missing teeth but also with enhancing facial aesthetics. Aging brings a lot of changes to the face, the most common being sunken cheeks. This article describes a simple, low-cost, and non-invasive method for the fabrication of non-detachable cheek plumper for edentulous patients. The two-in-one prosthesis not only replaces the missing teeth but also improves facial aesthetics.

## Introduction

Physical appearance plays an important role in the psychological wellbeing of a patient [1]. When young, many people try to pursue a sunken cheek look because it enhances the facial aesthetics. But the same look adds years to one's age as aging sets in. Aging brings a lot of changes in the body, especially the midface area, which is the most prominent region of our face. Aging is associated with the loss of teeth and supporting alveolar bone. This causes loss of facial fat and muscle tonicity, which leads to wrinkling and sagging of cheeks and lips [2]. Various treatment modalities have been mentioned in the literature for the treatment of sunken cheeks.

Cheek plumping prostheses should be considered when a patient is not interested in reconstructive surgery. A cheek plumper will lift the cheeks to correct contours without discomfort to the patient [3-9]. It will provide adequate support to sagging cheeks and lips. Various types of cheek plumpers have been mentioned in the literature. They can be broadly classified into detachable and non-detachable cheek plumper, each having its own merits as well as demerits.

This report describes a case of a completely edentulous patient rehabilitated with a non-detachable cheek plumper denture.

## Case presentation

A 70-year-old male patient presented to the department of prosthodontics for replacement of missing teeth due to difficulty in eating food. The patient also requested to improve the aesthetics of his sunken cheeks. History revealed that the patient was edentulous for two years.

On intra-oral examination, the patient had moderately resorbed maxillary and severely resorbed mandibular edentulous ridges. Extraoral examination revealed sunken cheeks. The patient was presented with all options, including fixed and removable cheek plumpers that were feasible, along with their pros and cons. The patient was allowed to make a conscious decision on the treatment that suited him best and written informed consent was acquired prior to its execution. The patient opted for a single prosthesis, that would serve the purpose of function and aesthetics. He didn't want the hassle of attaching the cheek plumper section after the insertion of the denture.

**Figure 1:**
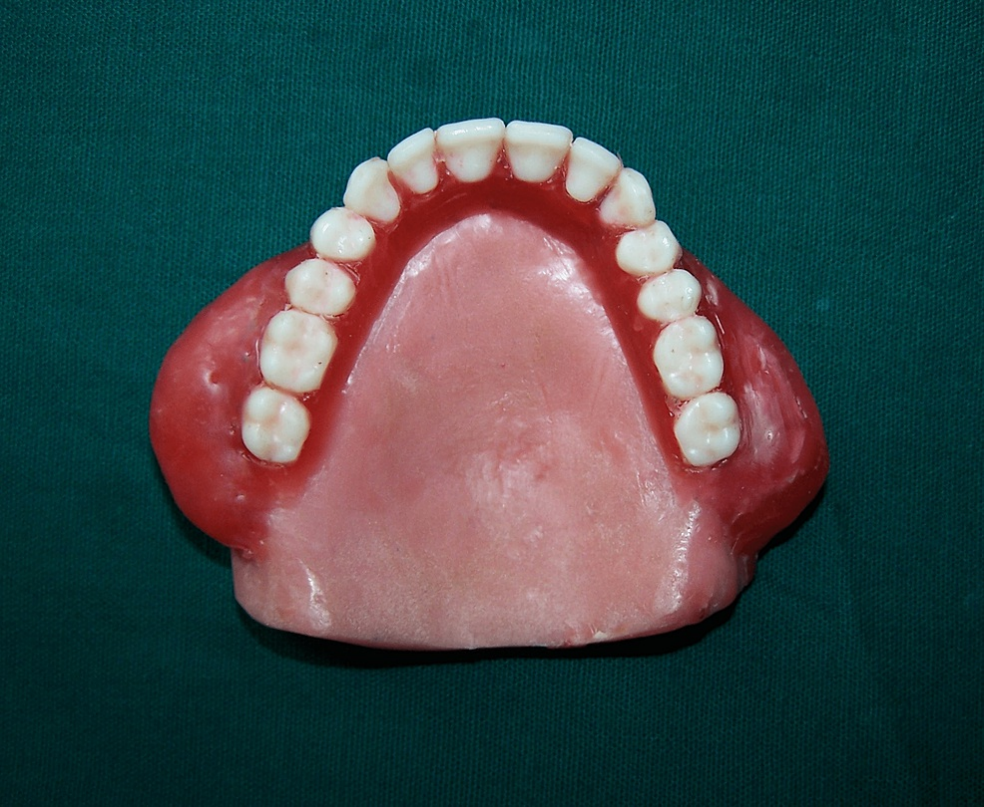
Wax-up of cheek plumper in trial denture

For both maxillary and mandibular dentures, preliminary impressions were made with an impression compound (DPI Pinnacle Impression Compound, Dental Products of India Ltd, Mumbai, India). After border molding of custom trays (DPI RR Cold Cure, Dental Products of India Ltd, Mumbai, India) using low fusion impression compound (DPI Pinnacle Tracing Sticks, Dental Products of India Ltd, Mumbai, India), definitive impressions were made with light body poly (vinyl siloxane) impression material (Express™, 3M Company, Saint Paul, Minnesota, United States). Jaw relations were recorded and mounted on a mean value articulator. The neutral zone was recorded using a soft tissue conditioner (Visco-Gel, Dentsply Sirona, Charlotte, North Carolina, United States). The same procedure was followed for recording the neutral zone as reported by Kursoglu et al. [10]. Teeth setting was done in neutral zone, which was obtained by the phonetic and functional method. During try-in, additional wax was added in the premolar molar region of the maxillary buccal flange (Figure 1).

Neutral zone was recorded by adding a thin layer of tissue conditioner separator on the waxed region of the plumper, followed by the application of tissue conditioner and asking the patient to perform functional movements. The adequate support and extent of cheek plumper were confirmed by extraoral appearance as well as neutral zone space. The patient's opinion about esthetics and comfort was also taken into consideration during the try-in of dentures.

**Figure 2:**
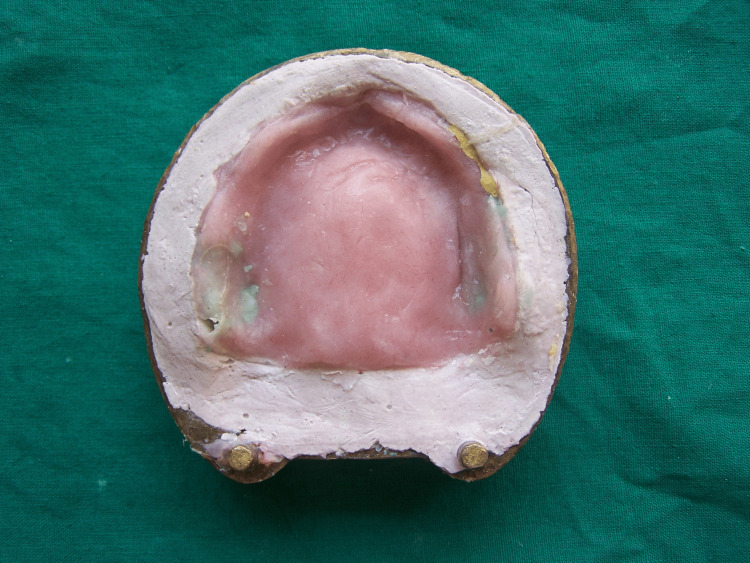
Trial closure with silicone putty material

After investing and dewaxing of dentures, a small amount of silicone putty impression material (Express, 3M Company, Saint Paul, Minnesota, United States) was placed into the cavity formed by the cheek plumper. Trial closure was carried out (Figure 2).

**Figure 3:**
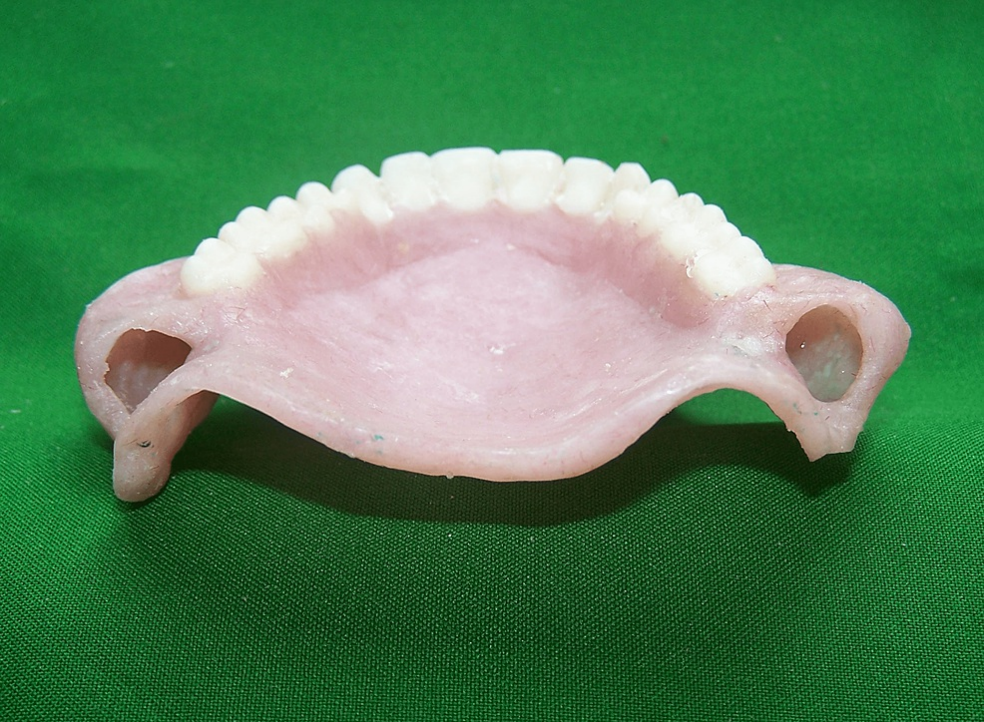
Hollow space in denture

The denture was processed according to the manufacturer’s instruction using heat polymerized acrylic resin (DPI Heat Cure, Dental Products of India Ltd., Mumbai, India) with a conventional compression molding technique. On retrieval of denture, silicone impression material was removed by creating a small hole with acrylic bur (Figure 3).

**Figure 4:**
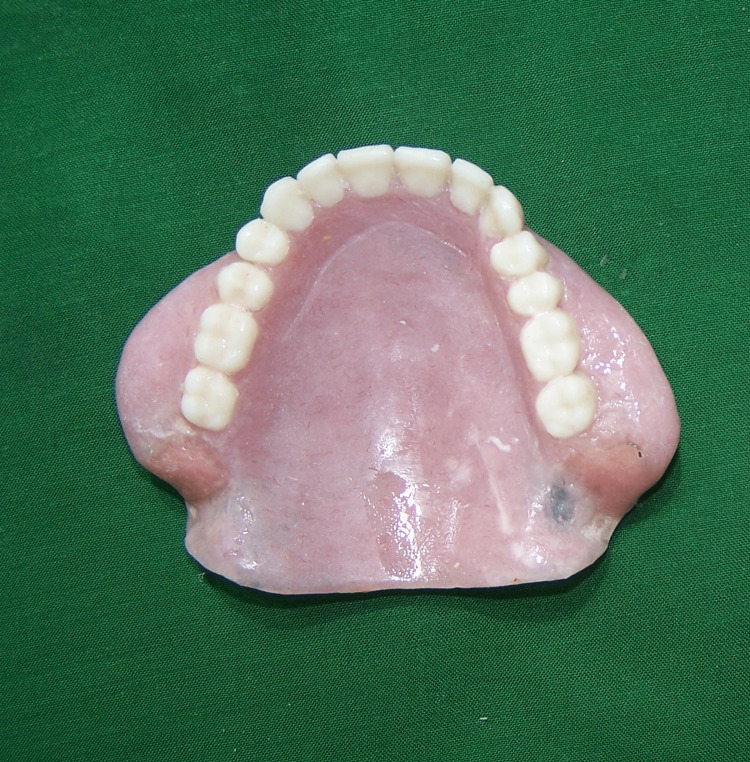
Denture with cheek plumper

The area was then repaired with auto polymerized acrylic resin (DPI RR Cold Cure, Dental Products of India Ltd, Mumbai, India). The denture was inserted into water and checked for any bubbles to verify for proper repair seal (Figure 4)

**Figure 5:**
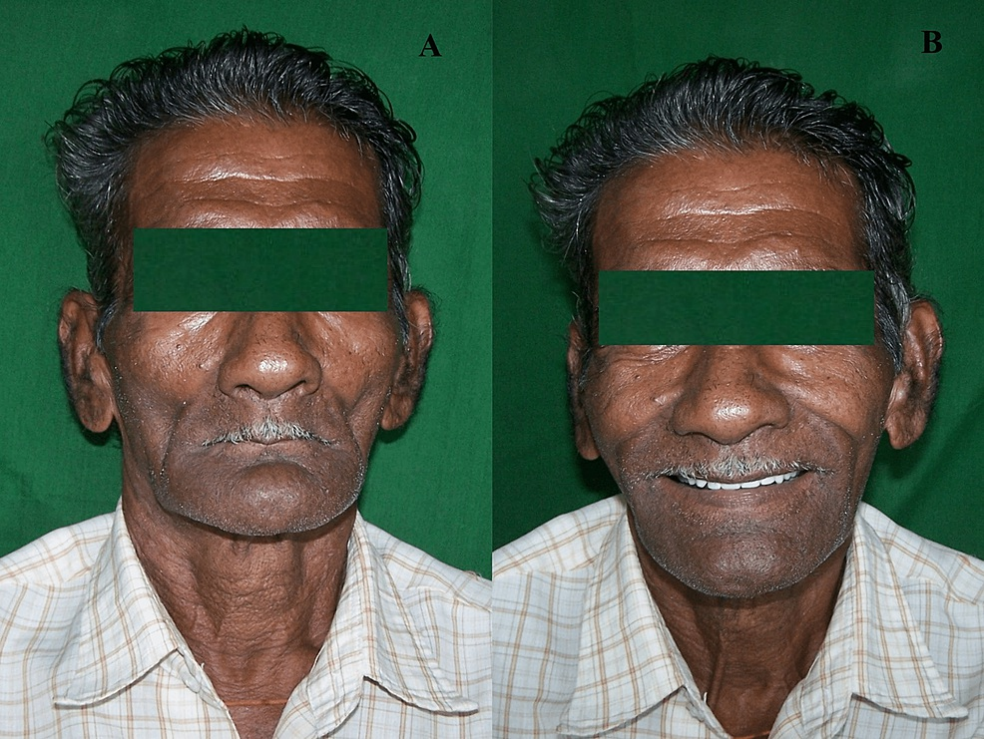
Pre-treatment (left) and post-treatment (right) images

**Figure 6:**
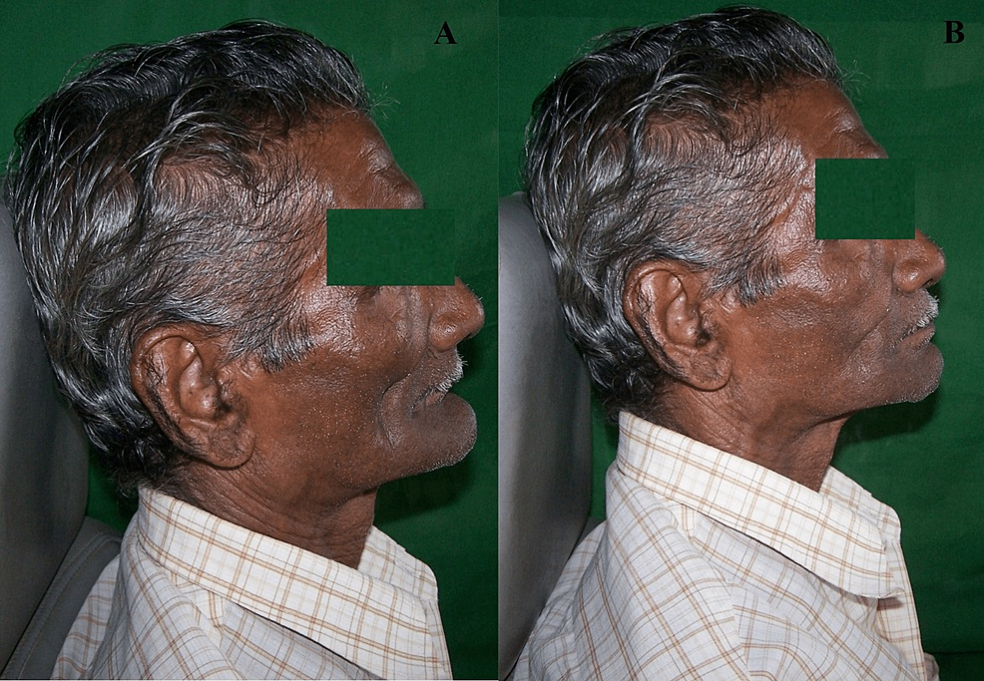
Pre-treatment (left) and post-treatment (right) images

The denture was delivered to the patient after proper finishing and polishing. The patient was recalled one day, three days, one week, and one month after insertion to check for function, aesthetics, comfort, and phonetics. The patient was also recalled three months, six months, and one year after insertion to check for any denture-related problems. The patient was very comfortable with the fit and comfort during recall visits and showed no signs of muscle fatigue with time (Figures 5, 6).

## Discussion

Facial appearance plays an important role in social as well as professional life. Sunken cheeks are easily visible giving the face an aged look. Various methods have been mentioned in the literature to enhance the esthetics of sunken cheeks. Dentists play an important role in the enhancement of facial esthetics by replacing not only missing teeth but also replacing surrounding structures. In an edentulous patient, this can be achieved by correct contours and extensions of denture flange. Sunken cheeks may require additional support to lift the cheek to an adequate level. Cheek plumper prosthesis can be an amazing solution for such patients [3-9]. Cheek plumper can be of detachable and non-detachable types. In detachable cheek plumper, the plumper is made separately and attached to the dentures with various attachments like magnets [3-6], die pin [7], snap button [8], and customized attachments [8,9]. Magnets tend to lose magnetic property with time and need to be re-magnetized [11]. In addition, they can contribute to corrosion if not encased in a stainless steel casing. Customized attachments will require additional time in fabrication. Snap buttons are not easily available in dental offices, and they need to be procured from outside. In the present case, non-detachable cheek plumper was fabricated from materials easily available in the dental office.

Cheek plumper was initially designed as single prosthesis attached to maxillary denture. This contributed to additional weight on the prosthesis and compromised the retention of denture. The added weight will also cause muscle fatigue with time. The patient's straightforward request was to make a non-detachable cheek plumper as he didn't want the hassle of attaching it after the insertion of denture. In the present case, hollow cheek plumper was planned for the patient. Since it was hollow, it didn’t add weight to the prosthesis and compromise the retention of the denture. Also, neutral zone technique contributed to the stability of denture. Silicon putty impression material was used to make hollow plumper. Putty impression material has advantage as it doesn’t adhere to acrylic resin material. It also can be easily retrieved from the denture.

For the denture to be in harmony with the stomatognathic system, the cheek plumper space was accessed using neutral zone technique. The limitation of this technique is that it cannot be used in microstomia patients because of the increased mediolateral width of dentures. The patient was recalled one day, three days, one week, one month, six months, and one year after insertion to check for function, aesthetics, comfort, and phonetics. The patient was very happy with ease of maintenance of denture hygiene as well as fit and contours of denture on recall visits.

## Conclusions

Simple and easy non-detachable cheek plumper denture was delivered to the edentulous patient with sunken cheeks. The cheek plumper not only improved facial esthetics of the patient but also enhanced his psychological wellbeing. It can be fabricated with materials easily available in dental setup.
